# Evaluation of treatment response in adults with relapsing MOG-Ab-associated disease

**DOI:** 10.1186/s12974-019-1525-1

**Published:** 2019-07-02

**Authors:** Alvaro Cobo-Calvo, María Sepúlveda, Fabien Rollot, Thais Armangué, Anne Ruiz, Elisabeth Maillart, Caroline Papeix, Bertrand Audoin, Helene Zephir, Damien Biotti, Jonathan Ciron, Francoise Durand-Dubief, Nicolas Collongues, Xavier Ayrignac, Pierre Labauge, Eric Thouvenot, Bertrand Bourre, Alexis Montcuquet, Mikael Cohen, Romain Deschamps, Nuria Solà-Valls, Sara Llufriu, Jerome De Seze, Yolanda Blanco, Sandra Vukusic, Albert Saiz, Romain Marignier

**Affiliations:** 1 Service de Neurologie, Sclérose en Plaques, Pathologies de la Myéline et Neuro-Inflammation, Hôpital Neurologique Pierre Wertheimer Hospices Civils de Lyon, Lyon, France; 2 Lyon Neuroscience Research Center, U1028 INSERM, UMR5292 CNRS, FLUID Team, 59 boulevard Pinel, 69677 Bron cedex, Lyon, France; 3 Center of Neuroimmunology, Service of Neurology, Hospital Clinic and Institut d’Investigació Biomèdica August Pi i Sunyer (IDIBAPS), University of Barcelona, Barcelona, Spain; 4 Faculté de Médecine Lyon-Est, Université Claude Bernard Lyon 1, Lyon, France; 5 Observatoire Francais de la Sclérose En Plaques (OFSEP), Hôpital Pierre-Wertheimer, Bron, France; 6 Pediatric Neuroimmunology Unit, Department of Neurology, Sant Joan de Deu Children’s Hospital, University of Barcelona, Barcelona, Spain; 7 Department of Neurology, Pitié-Salpêtrière Hospital, APHP, Paris, France; 8 Aix Marseille University, APHM, Hôpital de La Timone, Pôle de Neurosciences Cliniques, Service de Neurologie, Marseille, France; 9 Pôle des Neurosciences et de l’Appareil Locomoteur, CHU de Lille, Université de Lille, LIRIC, UMR 995, Lille, France; 10 Department of Neurology, Hôpital Pierre-Paul Riquet, University Hospital of Toulouse, Toulouse, France; 11 Department of Neurology and Clinical Investigation Center, Strasbourg University Hospital, Strasbourg, France; 12 Multiple Sclerosis Clinic, Montpellier University Hospital, Montpellier, France; 13 Department of Neurology, Hôpital Carémeau, Nimes University Hospital, Nimes, France; 14 Department of Neurology, Rouen University Hospital, Rouen, France; 15 Department of Neurology, Hôpital de Dupuytren, Limoges, France; 16 Université Côte d’Azur, Hôpital Pasteur 2, Centre Hospitalier Universitaire de Nice, Service de Neurologie, Nice, France; 17 Department of Neurology, Fondation A. De Rothschild, Paris, France; 18 Centre de référence des maladies inflammatoires rares du cerveau et de la moelle (MIRCEM), Lyon, France

**Keywords:** MOG antibodies, Treatment response, Neuromyelitis optica, Multiple sclerosis, Propensity score

## Abstract

**Background:**

Myelin oligodendrocyte glycoprotein antibodies (MOG-Ab) are related to several acquired demyelinating syndromes in adults, but the therapeutic approach is currently unclear. We aimed to describe the response to different therapeutic strategies in adult patients with relapsing MOG-Ab-associated disease.

**Methods:**

This is a retrospective study conducted in France and Spain including 125 relapsing MOG-Ab patients aged ≥ 18 years. First, we performed a survival analysis to investigate the relapse risk between treated and non-treated patients, performing a propensity score method based on the inverse probability of treatment weighting. Second, we assessed the annualised relapse rates (ARR), Expanded Disability Status Scale (EDSS) and visual acuity pre-treatment and on/end-treatment.

**Results:**

Median age at onset was 34.1 years (range 18.0–67.1), the female to male ratio was 1.2:1, and 96% were Caucasian. At 5 years, 84% (95% confidence interval [CI], 77.1–89.8) patients relapsed. At the last follow-up, 66 (52.8%) received maintenance therapy. Patients initiating immunosuppressants (azathioprine, mycophenolate mophetil [MMF], rituximab) were at lower risk of new relapse in comparison to non-treated patients (HR, 0.41; 95CI%, 0.20–0.82; *p* = 0.011). Mean ARR (standard deviation) was reduced from 1.05(1.20) to 0.43(0.79) with azathioprine (*n* = 11; *p* = 0.041), from 1.20(1.11) to 0.23(0.60) with MMF (*n* = 11; *p* = 0.033), and from 1.08(0.98) to 0.43(0.89) with rituximab (*n* = 26; *p* = 0.012). Other immunosuppressants (methotrexate/mitoxantrone/cyclophosphamide; *n* = 5), or multiple sclerosis disease-modifying drugs (MS-DMD; *n* = 9), were not associated with significantly reduced ARR. Higher rates of freedom of EDSS progression were observed with azathioprine, MMF or rituximab.

**Conclusion:**

In adults with relapsing MOG-Ab-associated disease, immunosuppressant therapy (azathioprine, MMF and rituximab) is associated with reduced risk of relapse and better disability outcomes. Such an effect was not found in the few patients treated with MS-DMD.

**Electronic supplementary material:**

The online version of this article (10.1186/s12974-019-1525-1) contains supplementary material, which is available to authorized users.

## Background

Myelin oligodendrocyte glycoprotein (MOG) antibody (Ab)-associated diseases are increasingly recognised as a distinct entity from either multiple sclerosis (MS) and aquaporin-4 (AQP4)-Ab-associated disease [1–7]. In adults, MOG-Ab has been found in patients with acquired demyelinating syndromes (ADS), including neuromyelitis optica spectrum disorders (NMOSD), limited forms related to the spectrum (optic neuritis [ON], transverse myelitis [TM]), encephalitis or brainstem syndromes [8–15].

Although initially MOG-Ab were mainly described in patients with a monophasic course with mild prognosis [1, 2, 4], recent studies reported a greater proportion of patients with a relapsing course and even a fulminant course with permanent disability [13, 16]. Moreover, whether relapses contribute to long-term disability in adults is under debate, since only a few studies have focused on relapsing patients and most of them included paediatric cohorts [17, 18].

Given that MOG-Ab-associated disease is a relatively new entity, physicians usually have some degree of uncertainty on how to manage these patients [2, 19]. Studies mixing paediatric and adult populations have shown that MOG-Ab-positive patients seem to be highly responsive to corticosteroids (CS) with an increased risk of relapse when tapering or following discontinuation [9, 16, 18]. However, long-term treatment with CS is limited by side effects underlying the need for steroid-sparing drugs. A recent study of paediatric patients with relapsing disease showed a reduction in relapse frequency associated with B cell-targeted therapies or intravenous immunoglobulins (IVIG) but not with MS disease-modifying drugs (MS-DMD) [17]. A beneficial effect of immunosuppressants (IS) and CS but not on MS-DMD has been reported in a case series [16]. However, more systematic studies in adults with MOG-Ab-associated disease dedicated to evaluate therapy strategies in real life have not been performed so far. In rare diseases such as NMOSD, clinical trials to measure treatment response are difficult to perform and the information is usually provided by observational studies [20, 21]. However, such studies are known to be influenced by potential bias. In this sense, the propensity score (PS) methods are the most common devices used to reduce bias when evaluating the effect of treatments on outcomes [22, 23].

We therefore conducted a retrospective multicentre study to describe the response to different therapeutic strategies used in real clinical practice in adults with relapsing MOG-Ab-associated disease.

## Methods

### Participants

We retrospectively recruited patients from all French and Spanish referral centres for neuroinflammatory disorders, within the scope of the *observatoire français de la sclérose en plaques* (OFSEP), and *Red Española de Esclerosis Múltiple* (REEM) that fulfilled the following inclusion criteria: (1) diagnosis of a relapsing ADS, defined as at least two acute clinical demyelinating episodes of the central nervous system (CNS) persisting for a minimum of 24 h; (2) age ≥ 18 years at onset of disease; (3) presence of MOG-Ab in serum and absence of aquaporin 4-Ab detected either at onset of disease or during follow-up.

### Clinical and therapeutic data

Clinical data already collected as part of both national programmes were de-identified, and merged in a new database. Epidemiological characteristics (sex, age at disease onset, ethnicity and country of provenience), clinical characteristics (phenotype at onset, date of conversion to NMOSD, severity at onset and last follow-up evaluated with the Expanded Disability Status Scale [EDSS]), imaging abnormalities (≥ 1 lesion on T2-weighted sequences) on the first brain magnetic resonance imaging (MRI), and cerebrospinal fluid (CSF) characteristics (cell count [pleiocytosis > 5 cells/mm^3^], oligoclonal bands [OCB] and IgG index) were included. For ON, visual acuity (VA) was evaluated by the visual functional system at the last follow-up in patients experiencing any ON.

At the end of the follow-up, patients were assigned to one of the following diagnostic categories; NMOSD-like phenotype fulfilling 2015 criteria [24], MS-like phenotype in those fulfilling McDonald 2010 criteria [25], relapsing ADS in a single CNS area (i.e. relapsing ON or TM) or multiphasic acute disseminated encephalomyelitis (MADEM) [24, 26]. Patients with short TM and ON who did not strictly fulfil NMOSD criteria were classified as optico-spinal phenotype [24].

Acute treatment such as oral or IV CS, plasma exchange (PLEX) or IVIG was noted at the first episode. Based on treatment experience [27], cumulative availability of clinical data as well as first- and second-line therapy recommendations [28], we classified azathioprine (AZT), mycophenolate mophetil (MMF) and rituximab (RTX) as type I IS, and cyclophosphamide (CYC), methotrexate (MTX) and mitoxantrone (MiTX) as type II IS. Long-term CS or IVIG was classified as type III IS [28]. Beta-interferon, glatiramer acetate, teriflunomide, natalizumab or fingolimod was classified as MS-DMD. Treatment regimens are depicted in Additional file 1: Table S1).

Based on pharmacodynamics and previous treatment experience, patients treated for at least 6 months were included in the treated group, and if not, they were included in the non-treated group. Reason for discontinuing treatment was also collected. In this retrospective study, the choice of treating was based upon the neurologists’ choice.

### Cell-based assays

AQP4-Ab and MOG-Ab tests were performed in the Lyon Neuroscience Research Center (France) and the Institut d’Investigació Biomèdica August Pi i Sunyer of Barcelona (Spain), by live cell-based assays (CBA) and using the protocols and plasmids as reported elsewhere [8, 29].

### Statistical analysis

We first described the clinical features of the total cohort. To describe probabilities of first relapse or NMOSD conversion in the whole cohort and according to clinical phenotype at the onset, we performed Kaplan-Meier survival analysis (with 95% of confidence interval, 95%CI) using time from onset of disease (first episode) to first relapse or NMOSD conversion.

**Fig. 1:**
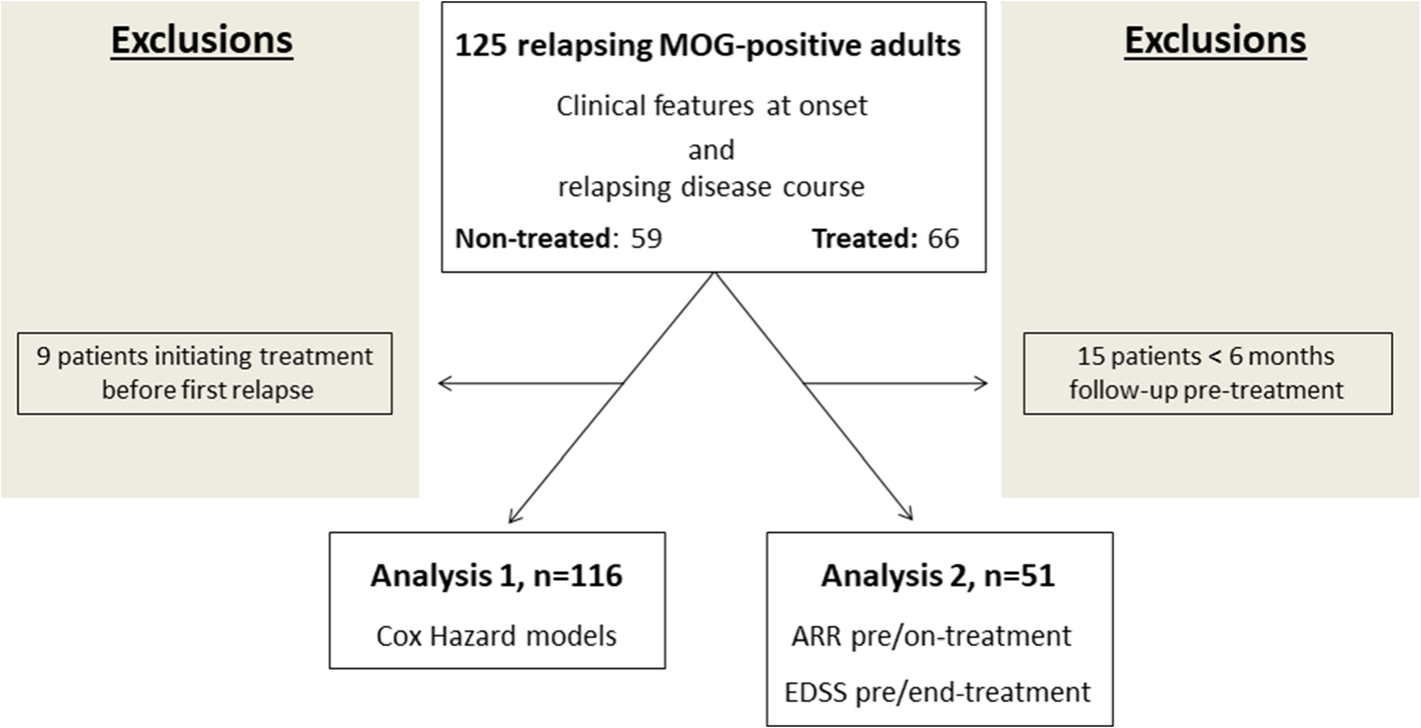
Flow chart of relapsing MOG-Ab adult patients included in different analyses

To evaluate treatment response, we considered two statistical methods:

Analysis 1. In order to study the effectiveness of treatments in a group of comparable patients and to limit treatment-related indication bias, we defined an ambivalence clause that allowed us to create a baseline date at which all patients had the opportunity to receive treatment. We assumed that all patients at diagnosis of relapsing ADS (at first relapse) were likely to receive treatment. Thus, we defined the baseline date (T0) as the date of treatment initiation for treated patients and as the date of relapsing ADS diagnosis (date of first relapse) for non-treated patients. We eliminated from the analysis patients initiating treatment before diagnosis of relapsing ADS in order to reduce a possible underestimation bias on treatment effect (Fig. 1). To measure the effect of treatments, the following possible confounders were taken into consideration: sex, age at onset, ethnicity, time between onset and the first relapse, phenotype at onset, EDSS at nadir, abnormal first brain MRI and country of provenience. Only confounders with *p* value < 0.20 were included in the construction of the PS model. The PS method based on the inverse probability of treatment weighting (IPTW) was used to estimate confounder-adjusted absolute risks in both treated and non-treated groups. This method balances the two groups to make them comparable across all confounders. With this approach, we modelled how the probability of receiving treatment depends on the confounders. For each patient, the PS was the individual predicted probability to receive the treatment according to baseline confounding variables, and obtained by binary logistic regression using the treatment group as outcome. The weight was the ratio between the mean probability to receive the treatment and the individual predicted probability to receive this treatment. Then, a weighted Cox proportional hazards model was used to estimate the effect of treatment on the outcome. To compare different treatments to non-treated patients, we calculated a PS for each comparison [22, 23]. For the analysis, an intention-to-treat strategy was used.

Analysis 2. Pre-treatment and on-treatment annualised relapse rates (ARR) for each patient were calculated (after excluding the index event). EDSS pre-treatment (the closest EDSS to the initiation date of treatment and sustained for at least 6 months) and end-treatment (the closest EDSS to end of treatment date and sustained for at least 6 months) were also evaluated. Similarly, VA pre-treatment and end-treatment were also noted. We exclude any transient worsening of disability related to relapses when measuring EDSS or VA. ARR, EDSS and VA were calculated for patients with at least 6 months of follow-up (Fig. 1).

If the same drug was given recurrently with a latency of > 3 months between 2 cycles, only the time from the first to the last drug application of the first cycle was considered and the other cycles with the same drug were rejected. This was observed for AZT, MMF and MS-DMD. For the other treatments, no recurrent treatment episodes were observed with the exception of one patient treated with type II IS (CYC followed by MTX 1 year apart). In this case, the first treatment period was considered for the analysis.

Wilcoxon’s matched-pairs rank sum test was used to compared ARR, EDSS and VA pre-treatment and on/end-treatment, and results were given as mean and standard deviation (SD). Only treatments with ≥ 5 patients who fulfilled the inclusion criteria were eligible for the analysis.

All statistical analyses were performed using STATA-12 (64-bits) software and a *p* value < 0.05 was considered significant. Graphs were constructed with GraphPad Prism (version 5.0) or R-3.4.4. Due to the exploratory nature of the study, we did not correct for multiple comparisons in either of the analyses.

## Results

### Cohort description

#### Clinical features at first episode

We identified 125 patients with relapsing MOG-Ab-associated disease. Median age at onset was 34.1 years (range 18.0–67.1), and the median duration of disease was 4.5 years (interquartile range [IQR], 1.8–10.2). Patients were mainly Caucasian (*n* = 120, 96.0%) with a female to male ratio of 1.2:1.0. Clinical phenotype at onset was characterised by ON in 82 (65.6%) patients, myelitis in 25 (20%) and ON together with myelitis in 9 (7.2%). Encephalopathic or brainstem syndromes were found in 9 (7.2%) patients. Among paraclinical features, 41/98 (41.8%) had pleiocytosis and 10/107 (9.4%) had OCB in the CSF. The first brain MRI showed abnormalities in 28/74 (37.8%) patients.

The diagnosis at the last follow-up was relapsing ON in 61 (48.8%) patients, NMOSD-like phenotype in 41 (32.8%) and relapsing TM in 11 (8.8%; 5 patients had extensive TM [LETM]), optico-spinal phenotypes in 4 (3.2%) and relapsing brainstem syndrome in 2 (1.6%). MADEM (all with ON relapses; ADEM-ON) was diagnosed in 3 (2.4%) patients, and MS-like phenotype in 3 (2.4%) patients (Table 1 and Additional file 1: Table S2 for features of MS patients).

**Table 1:** Epidemiological and clinical features according to diagnosis at last follow-up

	Total population *n* = 125	Relapsing ON *n* = 61	NMOSD-like phenotype *n* = 41	^b^Relapsing TM *n* = 11	^c^MADEM/brainstem S *n* = 5	^d^MS-like/optico-spinal phenotype *n* = 7
Females, *n* (%)	69 (55.2)	33 (54.1)	24 (58.5)	5 (45.5)	3 (60)	4 (57.1)
Age at onset, years, median (range)	34.1 (18.0–67.1)	36.0 (18.0–67.1)	34.6 (18.0–62.5)	33.7 (18.0–42.1)	45.7 (31.3–60.7)	22.7 (19.4–53.7)
Caucasian, *n* (%)	120 (96)	58 (95.1)	40 (97.6)	11 (100)	5 (100)	6 (85.7)
Follow-up, years, median (range)	4.5 (0.2–47)	1.4 (0.4–47)	5.7 (0.2–47)	10.9 (2.1–21.2)	2.5 (0.56–4.0)	5.5 (0.2–19.3)
Phenotype at onset, *n* (%)						
ON	82 (65.6)	61 (100)	16 (39)	0	0	5 (71.4)
Myelitis	25 (20)	0	12 (29.3)	11 (100)	0	2 (28.6)
ON and myelitis	9 (7.2)	0	9 (22)	0	0	0
Encephalopathic/brainstem S.	9 (7.2)	0	4 (9.7)	0	5 (100)	0
EDSS at onset, median (range)	3 (0–9)	2.5 (0–4)	3.25 (0–7.5)	3 (1–6)	4.5 (3.5–9)	3.5 (3–8)
EDSS 0–2.5	47 (38.2)	30 (50)	22 (55)	4 (36.6)	3 (60)	5 (71.4)
EDSS 3–5.5	64 (52)	30 (50)	32 (55)	4 (36.6)	3 (60)	5 (71.3)
EDSS ≥ 6.0	12 (9.8)	0	6 (15)	2 (18.2)	2 (40)	2 (28.6)
^a^ARR mean (SD)	0.79 (0.91)	0.80 (0.76)	0.64 (0.76)	0.46 (0.41)	1.13 (1.06)	1.78 (2.19)
Acute treatment MTP/PLEX/IVIG), *n* (%)	120 (96)	61 (100)	39 (95.1)	10 (90.9)	3 (60)	7 (100)
Paraclinical features, *n* (%)						
CSF OCB	10/107 (9.4)	1/49 (2.04)	4/36 (11.1)	3/11 (27.3)	0/5 (0)	2/6 (33.3)
CSF pleiocytosis	41/98 (41.8)	7/46 (15.2)	21/31 (67.7)	6/10 (60)	4/5 (80)	3/6 (50)
Abnormal brain MRI, at onset	28/74 (37.8)	7/38 (18.4)	11/23 (47.8)	3/6 (50)	4/4 (100)	3/3 (100)
EDSS at the last follow-up, median (range)	2 (0–7)	1.0 (0–4)	2 (0–7)	2 (0–4)	2.5 (1–3.5)	3.5 (0–6.5)
EDSS 0–2.5	86 (69.9)	48 (80)	26 (65)	7 (63.6)	3 (60)	2 (28.6)
EDSS 3–5.5	31 (25.2)	12 (20)	11 (27.5)	4 (36.6)	2 (40)	2 (28.6)
EDSS ≥ 6.0	6 (4.9)	0	3 (7.5)	0	0	3 (42.9)
VA at the last follow-up						
VA ≥ 0.7	61/100 (61)	36/61 (59)	18/31 (58.1)	–	3/3 (100)	4/5 (80)
VA > 0.2–0.6	21/100 (21)	13/61 (21.3)	8/31 (25.8)	–	0	0
VA ≤ 0.2	18/100 (18)	12/61 (19.7)	5/31 (16.1)	–	0	1/5 (20)

^a^For ARR (SD), index event was excluded

^b^Five patients had an extensive transverse myelitis

^c^Three patients had multiphasic-ADEM with further ON relapses (ADEM-ON)

^d^Optico-spinal phenotypes in 4, multiple sclerosis-like phenotype in 3 patients

*ON* optic neuritis, *NMOSD* neuromyelitis optica spectrum disorder, *TM* transverse myelitis, *ADEM-ON* acute disseminated encephalomyelitis-optic neuritis, *Brainstem S* brainstem syndrome, *MS* multiple sclerosis, *Optico-spinal* optico-spinal phenotype, *EDSS* Expanded Disability Status Scale, *ARR* annualised relapse ratio, *SD* standard deviation, *MTP* methylprednisolone, *PLEX* plasma exchange, *IVIG* intravenous immunoglobulins, *CSF* cerebrospinal fluid, *OCB* oligoclonal bands, *MRI* magnetic resonance imaging, *VA* visual acuity

#### Clinical course of disease

**Fig. 2:**
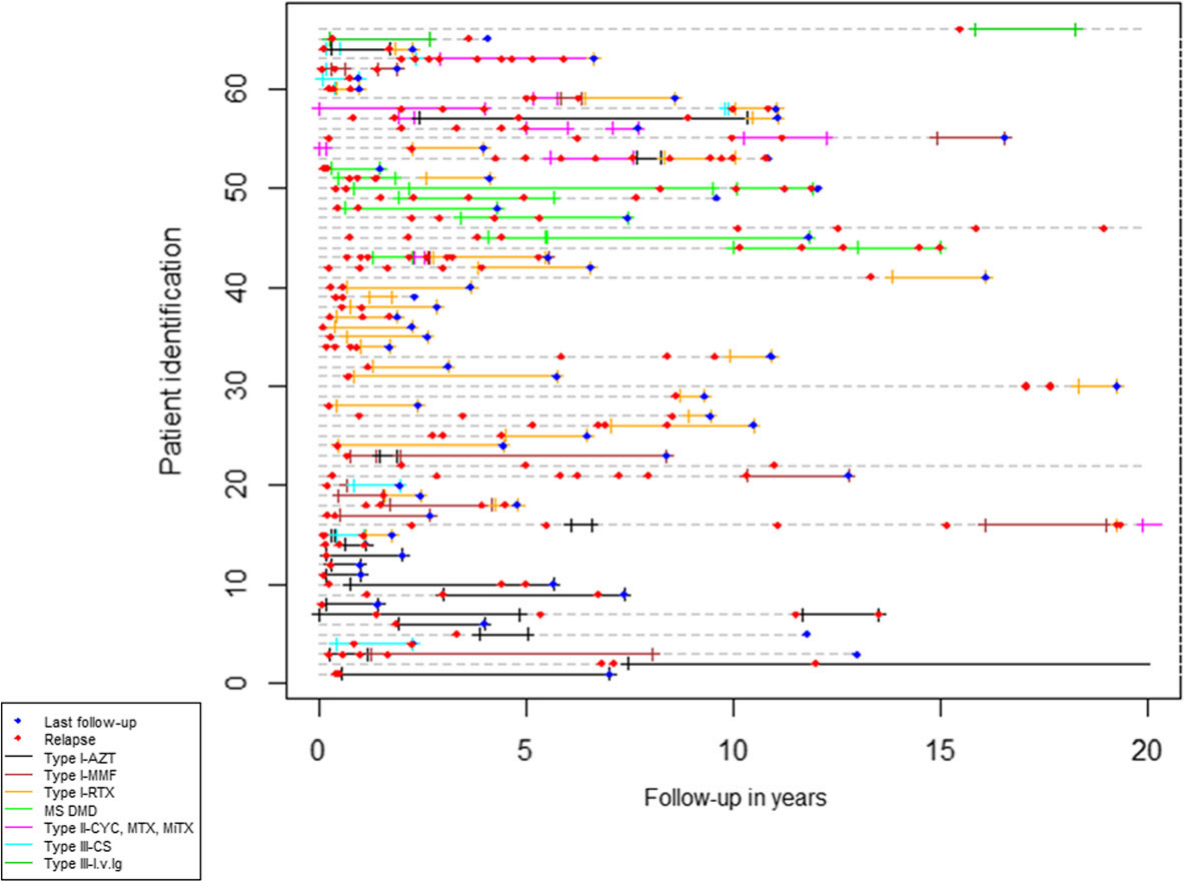
Relapsing disease course in the 66 treated patients from the total cohort. AZT, azathioprine; MMF, mycophenolate mophetil; RTX, rituximab; MS-DMD, multiple sclerosis disease-modifying drugs; CYC, cyclophosphamide; MTX, methotrexate; MiTX, mitoxantrone; CS, corticoids; i.v.Ig, intravenous immunoglobulins. *Patients Id.4, Id.20, Id.60, Id.62 and Id.63 followed therapies in combination (detailed in (Additional file 1: Table S5). **Treatment information in patients Id.2, Id.22, Id.47 and Id.66 is not depicted when started 20 years from the onset of symptoms

One hundred twenty (96%) patients received acute treatment at the onset. At last follow-up, 66 (52.8%) patients received maintenance therapy ≥ 6 months at some point; 47 (71.2%) patients were treated with one treatment, 15 (22.7%) with two treatments and 4 (6.1%) with three treatments. Among the 66 treated patients, only 9 (13.6%) initiated maintenance therapy before the first relapse (Fig. 2). EDSS at onset from these 9 patients did not differ from patients starting maintenance therapy after the first relapse (*p* = 0.175). At the first episode, PLEX and IVIG were more often prescribed in patients who further received maintenance therapy than in those without such treatment (16/66 [24.2%] vs. 3/59 [5.1%]), respectively, *p* = 0.003).

The majority of patients had good recovery at the last follow-up; 86 (69.9%) had mild (EDSS ≤ 2.5), 31 (25.2%) moderate (EDSS 3–5.5) and 6 (4.9%) severe disability (EDSS ≥ 6.0). Sixty one out of 100 (61%) patients with ON during the disease course had mild (VA ≥ 0.7), 21 (21%) moderate (0.2–0.6) and 18 (18%) severe VA disability at the last follow-up (Table 1).

A total of 438 demyelinating events were reported. The median number of relapses was 2 (IQR, 1–3). Patients who presented ≥ 2 relapses had higher EDSS at the last follow-up (median 2 [IQR, 1–3] than those with 1 relapse (median 1.5 [IQR 0–3]), *p* = 0.022, (Wilcoxon signed-rank test).

**Fig. 3:**
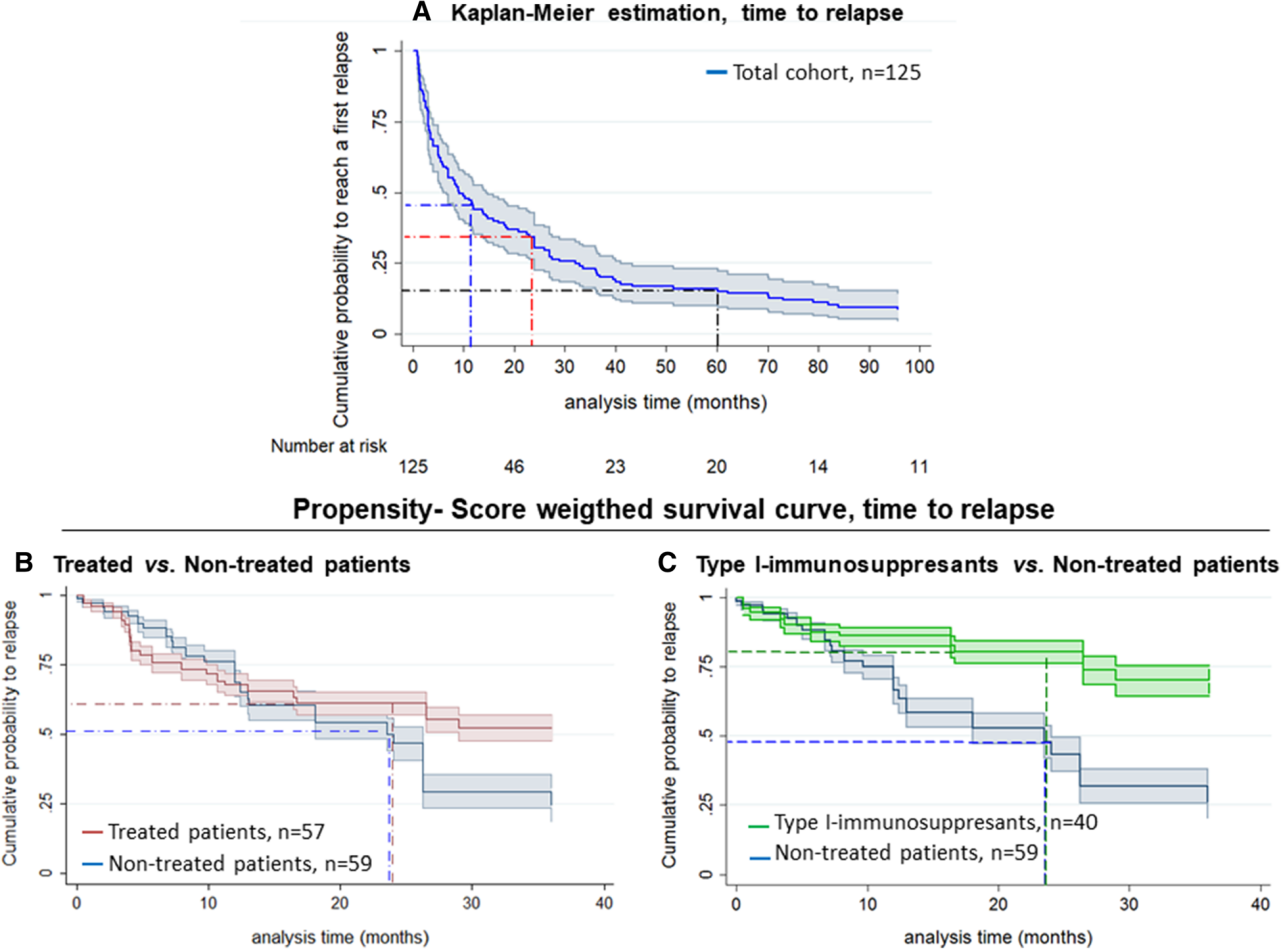
Kaplan-Meier analysis. **a** Time to relapse in the whole cohort; blue, red and black discontinuous lines represent the estimated probability of relapsing after 1, 2 and 5 years, respectively. The 95% confidence interval is shown in grey. **b**, **c** Propensity score weighted-survival curves, according to treatment. *The slight difference between the two analyses for the non-treated group is due to the different PS used for each model

Within the first year, 56% (95%CI, 47.6–64.8) of patients relapsed. At 2 years, 68% had relapsed (95%CI, 59.8–76.0), and at 5 years, 84% had done so (95%CI, 77.1–89.8) (Fig. 3a). Clinical phenotype at onset of symptoms was not related to relapse risk (Additional file 2: Figure S1A). However, at 2 years a greater proportion of patients presenting with TM at onset were diagnosed with NMOSD (38.2% [95%CI, 20.8–63.0]) than ON (17.3% [95%CI, 8.9–32.4]; Log-rank *p* = 0.032; Additional file2; Figure S1B).

### Treatment responses

#### Analysis 1: relapse risk according to treatment

Comparison of baseline possible confounders between treated and non-treated patients in the original sample and the pseudo-population weighting by PS are shown in (Additional file 1: Table S3 and S4).

PS-weighted survival analysis found that the 2-year risk of relapse was 49.9% (95%CI, 44.2–56.0) for the 59 non-treated patients compared to 38.6% (95%CI, 34.6–42.9) for the 57 treated patients (Fig. 3b). The 2 year-risk of relapse was 52.1% (95%CI, 46.3–58.1) for the 59 non-treated patients compared to 19.4% (95%CI, 15.7–23.9) for the 40 type I IS-treated patients (Fig. 3c). The slight difference between the two analyses for the non-treated group is due to the different PS used for each model.

Although relapse rate was significantly lower in treated than in non-treated patients (HR, 0.58; 95%CI, 0.34–0.99; *p* = 0.050) when performing the crude analysis, the difference was no longer significant after PS weighting (HR, 0.68; 95%CI, 0.40–1.16; *p* = 0.155). Type I IS-treated patients were at significantly lower risk of relapse (HR, 0.40; 95%CI, 0.21–0.77; *p* = 0.006) than non-treated patients, and this difference was still present after PS weighting (HR, 0.41; 95CI%, 0.20–0.82; *p* = 0.011; Table 2). PS-weighted proportional hazards Cox models were not used in the other groups due to the low number of patients.

**Table 2:** Distribution of relapse risk in non-treated and treated patients, according to treatment strategies

Variables	Non-treated *N* = 59	^a^Treated *N* = 57	Treatment strategy
			Type I IS *N* = 40
Patients with clinical relapse, *n* (%)	28 (47.5)	27 (47.4)	14 (35.0)
Time from first relapse to treatment, months, median (range)	–	5.0 (0–532.6)	5.1 (0–532.6)
^b^Time from T0 to relapse, months, median (range)	7.9 (0.1–84.2)	15.1 (0.5–211.1)	21.9 (0.5–94.1)
Treatment duration, months, median (range)	–	22.3 (6.0–176.1)	22.2 (6.0–151.0)
HR, crude (95%CI)	–	0.58 (0.34–0.99), *p* = 0.050	0.40 (0.21–0.77), *p* = 0.006
HR, propensity score (95%CI)	–	0.68 (0.40–1.16), *p* = 0.155	0.41 (0.20–0.82) *p* = 0.011

^*^PS-weighted proportional hazards Cox models were not used in the other groups due to the low number of patients

^a^Among treated patients (*n* = 57), 9 patients were excluded since started treatment before the first relapse

^b^T0 was defined as the date of treatment initiation for treated patients and as the date of relapsing ADS diagnosis (date of the first relapse) for non-treated patients

*HR* hazard ratio, *CI* confidence interval, *IS* immunosuppressants

Three out of 15 (20%) patients starting with AZT, 0 out 6 patients with MMF and 2 out of 17 (10.5%) starting with RTX relapsed within the first 6 months after starting respective treatments.

#### Analysis 2: annualised relapse ratio and disability according to treatments

Overall, 49/66 (74.2%) treated patients received AZT, MMF, or RTX (≥ 6 months each) at any time.

#### Azathioprine

AZT was given to 19/66 (28.8%) treated patients at any time; 15 of them (78.9%) received AZT as first-line therapy and 3 (15.8%) as second-line. Nine (47.4%) patients discontinued AZT: 4 for general or biological intolerance, 3 for physician or patient decision and 2 for treatment failure. AZT (*n* = 11 eligible for analysis) was associated with a reduction of the mean ARR from pre-treatment, 1.05 (1.20), to on-treatment, 0.43 (0.79) (*p* = 0.041), and there was no difference between pre-treatment EDSS and end-treatment (*p* = 0.157). While on AZT, 6 (54.5%) patients remained freedom of relapse, and 11 (100%) freedom of EDSS progression (Tables 3 and 4). One patient (9%) relapsed at 5.6 months after starting AZT.

**Table 3:** Evaluation of pre-treatment and on-treatment annualised relapse ratio and EDSS according to treatment group

Treatment group		Treated ≥ 6 months at any time, *n* (%)	Eligible for analysis, *n* (%)	FU before treatment (years), median (range)	FU under treatment (years), median (range)	ARR pre/on-treatment, mean (SD)	Freedom of relapse on-treatment *n* (%)	*p* value ARR pre/on-treatment	EDSS pre/end of treatment, mean (SD)	Freedom of EDSS progression, *n* (%)	*p* value EDSS pre/end-treatment
Type I IS	AZT	19/66 (28.8)	11/19 (57.9)	2.4 (0.6–7.6)	2.1 (0.5–12.6)	1.05 (1.20)/ 0.43 (0.79)	6 (54.5)	0.041	1.86 (1.30)/ 1.68 (1.19)	11 (100)	0.157
	MMF	12/66 (18.2)	11/12 (91.7)	1.7 (0.5–46.4)	1.7 (0.5–6.8)	1.20 (1.11)/ 0.23 (0.60)	8 (72.7)	0.033	2.72 (1.69)/ 2.64 (1.76)	11 (100)	0.317
	RTX	30/66 (45.5)	26/30 (86.7)	3.3 (0.5–18.33)	1.7 (0.5–4.9)	1.08 (0.98)/ 0.43 (0.89)	19 (73.1)	0.012	3.11 (1.83)/ 2.58 (1.90)	23 (88.5)	0.096
Type II IS		6/66 (9.1)	5/6 (83.3)	5.2 (2.9–10.3)	2.0 (0.6–3.7)	0.64 (0.45)/ 0.65 (0.69)	2 (40)	0.893	3.8 (1.52)/ 4.0 (1.45)	1 (20.0)	0.317
Type III IS		8/66 (12.1)	3/8 (37.5)	–	–	–	–	–	–	–	–
^a^MS-DMD		10/66 (6.6)	9/10 (90)	1.95 (0.5–20.1)	3.7 (1.0–14.7)	1.13 (1.38)/ 0.49 (0.41)	2 (22.2)	0.374	2.5 (0.90)/ 3.17 (2.15)	7 (77.7)	0.188

*Patients treated with type III IS (corticosteroids, *n* = 2 and intravenous immunoglobulins, *n* = 1) were not eligible for analysis due to treated number ≤ 5

^a^Among the 9 patients with MS-DMD eligible for the analysis, 2 patients were treated with natalizumab, 1 with glatiramer acetate and 6 with interferon

*FU* follow-up, *ARR* annualised relapse ratio, *SD* standard deviation, *EDSS* Expanded Disability Status Scale, *IS* immunosuppressants, *MS-DMD* multiple sclerosis disease-modifying drugs, *AZT* azathioprine, *MMF* mycophenolate mophetil, *RTX* rituximab

**Table 4:** Treatment options in different groups

	Type I-IS *N* = 61			^a^Type II IS *N* = 6	Type III IS *N* = 8	^b^MS-DMD *N* = 10
	AZT *N* = 19	MMF *N* = 12	RTX *N* = 30			
Therapy choice, *n* (%)						
First line	15 (78.9)	6 (50)	19 (63.3)	5 (83.3) (3 CYC, 1 MTX, 1 MiTX)	5 (62.5) (3 CS, 2 IVIG)	10 (100) (6 IFN, 1 TFN, 1 GA, 2 NTZ)
Second line	3 (15.8)	5 (41.7)	5 (16.6)	1 (16.6) (1 MTX)	3 (37.5) (3 CS)	–
Other lines	1 (5.3)	1 (8.3)	6 (29.4)		–	–
Patients discontinuing treatment, *n* (%)	9 (47.4)	7 (58.3)	3 (10)	6 (100)	5 (55.6)	7 (70)
Causes for discontinuing treatment, *n* (%)						
General intolerance	2 (22.2)	1 (14.3)	1 (33.3)	–	–	1 (14.3)
Biological intolerance	2 (22.2)	–	–	1 (16.7)	–	–
Physician decision	2 (22.2)	2 (28.6)	1 (33.3)	3 (50)	4 (80)	1 (14.3)
Patient decision	1 (11.1)	–	–	–	–	–
Treatment failure	2 (22.2)	4 (57.1)	1 (33.3)	2 (33.3)	1 (20)	4 (57.1)
Pregnancy desire	–	–	–	–	–	1 (14.3)

^a^ One patient switched from CYC to MTX

^b^Three patients switched from MS-DMD to another MS-DMD

*IS* immunosuppressants, *MS-DMD* multiple sclerosis disease-modifying drugs, *AZT* azathioprine*, MMF* mycophenolate mophetile, *RTX* rituximab, *CS* corticoids, *CYC* cyclophosphamide, *IVIG* intravenous immunoglobulins, *MTX* methotrexate, *MiTX* mitoxantrone, *IFN* interferon, *TNF* teriflunomide, *GA* glatiramer acetate, *NTZ* natalizumab

#### Mycophenolate mophetil

Twelve (18.2%) out of 66 treated patients received MMF; 6 of them (50%) received MMF as first-line therapy, and 5 (41.7%) as second-line. MMF was discontinued in 7 (58.3%) patients; 1 for general intolerance, 2 for physician decision and 4 for treatment failure. MMF (*n* = 11 eligible for analysis) was associated with a reduction in the mean ARR from 1.20 (1.11) to 0.23 (0.60) (*p* = 0.033), and no changes in the EDSS were observed (*p* = 0.317). While on MMF, freedom of relapse was found in 8 (72.7%) patients, and freedom of EDSS progression in 11 (100%) (Tables 3 and 4). Two patients (16.6%) relapsed at 5 and 4.7 months after starting MMF, respectively.

#### Rituximab

Thirty (45.5%) out of 66 treated patients received RTX; 19 (63.3%) of them received RTX as first-line therapy, and 5 (16.6%) as second-line. Only one patient discontinued the therapy due to general intolerance. Physician decision and treatment failure was the reason for discontinuing RTX in the other two patients. The mean ARR was reduced from 1.08 (0.98) to 0.43 (0.89) with RTX (*n* = 26 eligible for analysis), *p* = 0.012. Freedom of relapse on RTX was observed in 19 (73.1%) patients and freedom of EDSS progression in 23 (88.5%) (Tables 3 and 4). Among the seven patients who relapsed, three patients (11.5%) relapsed at 1.7, 3 and 3.4 months after starting the first infusion of RTX, respectively, and one patient at month 5 after the last infusion.

*Type II* or *type III IS* was given to 14 (21.2%) treated patients; type II IS as first-line in 5 (83.3%) and type III IS in 5 (62.5%). As second-line therapy, 1 (16.6%) patient received type II IS with MTX, and 3 (37.5%) received type III IS with CS. Type II IS was discontinued in 6 (75%) patients, and type III IS in 5 (55.6%). Only patients with type II IS were eligible for analysis (*n* = 5), and we did not observe significant changes in ARR nor EDSS (Tables 3 and 4).

Five (7.6%) of treated patients followed a combination of CS with IS for a period of the disease (Additional file 1: Table S5).

*MS-DMD* was administered in 10 (6.6%) treated patients (all as first-line therapy). MS-DMD was discontinued in 7 (70%) patients; 4 for treatment failure and 1 for general intolerance, physician decision and pregnancy desire each. Three patients were switched to another MS-DMD, and two patients to RTX and MTX. Nine patients under MS-DMD were eligible for analysis (two patients were treated with natalizumab, one with glatiramer acetate and six with interferon) and did not show a significant reduction in the mean ARR or the EDSS (Table 3). Freedom of relapse was observed in 2 (22%) patients, and freedom of EDSS progression in 7 (77.7%) (Table 3).

Finally, there were no differences regarding the VA pre-treatment and end-treatment in type I IS; AZT (*n* = 9; *p* = 0.289), MMF (*n* = 10; *p* = 0.564) and RTX (*n* = 20; *p* = 0.157). VA analysis was not performed in type II, type III IS and MS-DMD subgroups since the number of patients experiencing ON during the disease course was lower than 5.

## Discussion

In this large cohort evaluating treatment response in MOG-Ab adult patients with relapsing course, we found that most patients relapsed soon after disease onset, and relapses were associated with a cumulative impact on long-term disability. Importantly, first-line therapies recommended by the international NMOSD guidelines had a favourable impact on clinical outcomes.

In keeping with other studies, relapses mainly occurred within the first year from onset of disease [9, 16]. Whether the cumulative disability is driven by poor recovery after onset or the relapsing course remains to be determined [9, 13]. Although our study shows an overall good prognosis, patients who had a higher frequency of relapse displayed worse disability at the last follow-up. This fact suggests that a cumulative effect given by the relapsing course may exist, underlying the need for a preventive therapy. Nonetheless, only 13.6% of treated patients received immunosuppressants after the onset of the disease and before the first relapse which likely reflects the current widespread perception about the benign course of the disease. We should note that our study deals with a cohort of exclusively MOG-Ab relapsing patients, and the information provided about the impact of relapses on disability is different from that observed in studies including monophasic and relapsing patients. Further studies designed to identify baseline prognostic factors are mandatory to select patients who will benefit from immunosuppression at the onset of the disease.

To date, there are no standardised international guidelines to manage MOG-Ab-associated disease, leading to heterogeneous policies not only regarding the type of maintenance therapy to use but also the time to initiate treatment [9, 16–18]. Our study shows that most physicians chose therapies included in the international guidelines for NMOSD [20, 21]. As recommended, RTX and AZT were the most widely prescribed IS, followed by MMF. Other IS such as CYC, MTX or MiTX, and MS-DMD were less frequently prescribed. Moreover, long-term CS or IVIG was not frequently used in French and Spanish routine clinical practice, contrary to recent trends encouraging their prescription due to the potential beneficial effect on decreasing relapses [9, 16, 18]. In fact, evidence from previous studies suggests that effectiveness of immunosuppressants may be more pronounced when patients are treated with oral CS during the latency period of treatments, usually during the first 6 months [16].

The main strength of the study lies in the combination of two statistical approaches in order to evaluate treatment response. First, we have controlled variables which may confound treatment assignment by using PS methods, thus, mitigating the effects of treatment indication bias. With this approach, we were able to evaluate response to the most frequently IS used in clinical practice (AZT, MMF, RTX) and we observed a reduction in the relapse risk when the patient is diagnosed with relapsing ADS. Repeated cycles of IGIV have shown to reduce relapses in children with relapsing ADS and MOG-Ab [17]. Although in the present study only a small proportion of patients were treated with IGIV/PLEX at the acute phase, we cannot completely exclude an impact of such treatment on the long-term outcome. Additionally, we performed more classical analysis to evaluate separately the effect of AZT, MMF and RTX on relapses, confirming their beneficial effect in reducing the ARR.

Although significant differences were obtained when comparing treated and non-treated groups after performing crude analysis, these differences were no longer significant in PS-weighted analyses. This example underlines the importance of using PS methods to balance treated and non-treated groups according to confounders otherwise biased results may be obtained.

AZT was mainly used as first-line therapy, while both MMF and RTX were less frequently selected as the first choice. Several observational studies have reported beneficial effects of both AZT and MMF over the clinical course in NMOSD [30–32]; more scarce information is available regarding MOG-Ab-associated disease. Recent data have shown an improvement in the ARR with the use of both drugs [17, 18], but special attention must be given in those patients not co-treated with corticoids during the latency period of the drugs due to the risk of breakthrough relapses [16]. RTX has increasingly been used both as first-line therapy and IS-unresponsive populations in NMOSD, leading to a sustained clinical stabilisation in most patients [33–35]. Although type I IS showed to be beneficial in decreasing ARR, none of them improved disability except for a trend with RTX. Doses of immunosuppressants may have an impact on outcomes and higher doses of AZT (2–5–3 mg/kg) are potentially associated to a better response than standard treatment (1–1.5 mg/kg) in AQP4-Ab-positive patients [30]. Herein, patients used standard protocol with a dose of 150 mg per day. It is noteworthy that the EDSS was evaluated at least 6 months after the relapse, and improvements in residual EDSS is less likely [17]. Moreover, the beneficial effect was also observed in the high figures of freedom in EDSS progression, and this fact is important taking into account the relatively high frequency of relapse of this population.

Adherence to AZT and MMF was poor, being discontinued in almost half of patients (Table 4). RTX was generally well tolerated (only one patient discontinued due to intolerance), and only two switched to another drug (MiTX and MMF, respectively). Potential severe adverse effects have been reported with RTX and, currently, the safety profile suggests being cautious to prescribe this drug as first-line therapy [36]. However, the present study was not designed to draw conclusions regarding drug tolerance.

A covariate balance to perform PS analysis between type II, type III IS or MS-DMD, and non-treated groups was not possible due to low sample numbers. However, with the second approach, we found that neither type II IS nor MS-DMD allowed controlling clinical activity or cumulative disability, as previously described for MS-DMD both in MOG-Ab-positive patients and NMOSD [17, 37–39]. Due to the low number of patients under MS-DMD in the present study, larger studies are needed to confirm our results. Nonetheless, our results are in line with other studies evaluating these drugs in MOG-Ab-associated disease [16, 17], suggesting a lack of effectiveness of MS-DMD. In contrast to the detrimental effect of MS-DMD in some patients with NMOSD [40], we did not observe such an effect in our population of adult patients with MOG-Ab.

Our study has limitations related to its retrospective nature and the lack of randomisation for treatment allocation. However, PS weighting was used to decrease indication bias. Moreover, an immortal time bias could be argued since type I IS-treated group could have presented the event before entry in the cohort (diagnosis of relapsing ADS) which could lead to an event overestimation in the non-treated group and, therefore, lower event rates in type I IS. Covariate balance was reached with PS analysis and, therefore, this immortal person-time period was properly addressed [41]. On the other hand, the cohort was not powered to analyse differences in every treated group due to the relatively small sample size. However, we performed two statistical methods to analyse the effectiveness of the most widely used treatments in this setting. Therapies were not prospectively controlled and patients switched treatments over time or had combined therapies without a washout period in some cases which may influence treatment effectiveness in terms of beneficial and harmful effects. We believe that a combination of therapies did not influence the overall results since only a few patients followed two treatments at the same time, allowing us to evaluate the effect of each IS.

## Conclusion

This study of a large cohort of patients with relapsing MOG-Ab-associated disease treated in real clinical practice provides several important observations: the better outcome in terms of relapses and disability for patients who are treated after having at least two episodes, and the beneficial effect of being treated with immunosuppressants such as AZT, MMF and RTX. In addition, the lack of effect in the patients treated with MS-DMD in this study highlights the importance of early identification of these patients with MOG-Ab although larger studies are needed to confirm such finding.

Overall, the present exploratory study found good response to type I IS, providing a rationale to investigate efficacy of these drugs. Randomised controlled trials are needed to obtain more definite data on optimum treatment in MOG-Ab-associated disease.

The data reported here, however, are only applicable for patients with relapsing MOG-Ab-associated disease.

## Additional files

Additional file 1: **Table S1.**Treatment regimens and intervals. Table S2 Characteristics in patients with MOG antibodies and multiple sclerosis. Table S3 Comparison of baseline possible confounders between treated and non-treated patients in the original sample. Table S4 Comparison of baseline possible confounders between treated and non-treated patients in the pseudo-population. Table S5 Patients who received combined drugs and reasons for inclusion/exclusion in different analyses. (DOCX 28 kb)

Additional file 2: **Figure S1. **(A) Kaplan- Meier estimation of time to first relapse, according to clinical phenotype at the onset. The 2-year risk of the first relapse was 70.7% (95%CI 60.7–80.1) for optic neuritis (reference), 64% (95%CI 45.8–81.8; Log-rank *p* = 0.589) for transverse myelitis, 77.8% (95%CI 48.7–96.6; Log-rank *p* = 0.458) for optic neuritis and transverse myelitis, and 44.4% (95%CI 19.6–79.6; Log-rank *p* = 0.617) for encephalopathy/brainstem syndrome. (B) Time to NMOSD-like phenotype conversion according to clinical phenotype at the onset. The 2-year risk to NMOSD-like phenotype conversion was 17.3%; 95%CI, 8.9–36.4 for optic neuritis (as a reference), 38.2% (95%CI, 20.8–63.0; Log-rank *p* = 0.032) for transverse myelitis, 14.3% (95%CI, 2.14–66.6; Log-rank *p* = 0.199) for encephalopathy/brainstem syndrome; 100% relapsed at onset (Log-rank *p* < 0.001) for optic neuritis and transverse myelitis (TIF 93 kb)

## Data Availability

This study was done within the framework of OFSEP. Because of national confidentiality requirements, only anonymized data, not pseudonymized data, can be shared. While anonymization techniques might result in the impoverishment of data (Article 29 of Directive 95/46/EC, Opinion 05/2014 on Anonymisation Techniques—0829/14/EN WP 216), data used for this study were only pseudonymized. However, access to OFSEP data to conduct a scientific project is possible by following the OFSEP data access process (ofsep.org/en/ data-access) and with respect to French law.

## Abbreviations

Ab: Antibody
ADS: Acquired demyelinating syndromes
AQP4: Aquaporin-4
ARR: Annualised relapse rates
AZT: Azathioprine
CBA: Cell-based assay
CNS: Central nervous system
CS: Corticosteroids
CSF: Cerebrospinal fluid
CYC: Cyclophosphamide
EDSS: Expanded Disability Status Scale
IS: Immunosuppressants
IVIG: Intravenous immunoglobulins
MADEM: Multiphasic acute disseminated encephalomyelitis
MiTX: Mitoxantrone
MMF: Mycophenolate mophetil
MOG: Myelin oligodendrocyte glycoprotein
MRI: Magnetic resonance imaging
MS: Multiple sclerosis
MS-DMD: MS disease-modifying drugs
MTX: Methotrexate
NMOSD: Neuromyelitis optica spectrum disorders
OCB: Oligoclonal bands
ON: Optic neuritis
PS: Propensity score
RTX: Rituximab
SD: Standard deviation
TM: Transverse myelitis
VA: Visual acuity
