# Supplementary material for: Evaluation of treatment response in adults with relapsing MOG-Ab-associated disease
Source: J Neuroinflammation. 2019 Jul 2;16:134. doi: 10.1186/s12974-019-1525-1 (PMC6607517; doi:10.1186/s12974-019-1525-1)
Supplement: Supplementary file 1 — Table S1.Treatment regimens and intervals. Table S2 Characteristics in patients with MOG antibodies and multiple sclerosis. Table S3 Comparison of baseline possible confounders between treated and non-treated patients in the original sample. Table S4 Comparison of baseline possible confounders between treated and non-treated patients in the pseudo-population. Table S5 Patients who received combined drugs and reasons for inclusion/exclusion in different analyses. (DOCX 28 kb) [file 12974_2019_1525_MOESM1_ESM.docx]

Additional file 1

Table S1.Treatment regimens and intervals

| **Type of IS** | **Treatment** | **Dosage** | **Administration** | **Interval** | | |
| --- | --- | --- | --- | --- | --- | --- |
| Type I-IS | AZT | 150 mg | Oral | daily | | |
|  | MMF | 2000 mg | Oral | daily | | |
|  | RTX | 1000 mg | IV | every 6 months | | |
| Type II-IS | CYC | 1000 mg | IV | monthly or every two months (Cumulative dose< 20 mg) |  |  |
|  | MTX | 7.5-12 mg | Oral | weekly | |  |
|  | MiTX | 12 mg/m^2^ | IV | every three months  (Cumulative dose < 120 mg) | |  |
| Type III-IS | Long-term CS | 1mg /kg | Oral | daily | |  |
|  | IVIG | 0.4 g/Kg | IV | daily x 5 days | |  |
| MS-DMD | B-Interferon  (1.a/1.b) | 30 mcg/ 250 mcg | IM/SC | Alternate days | |  |
|  | Glatiramer acetate | 20mg | SC | daily | |  |
|  | Teriflunomide | 14 mg | Oral | daily | |  |
|  | Natalizumab | 300 mg | IV | monthly | |  |
|  | Fingolimod | 0.5 mg | Oral | daily | |  |

*IS* immunosuppressants; *AZT* azathioprine; *MMF* mycophenolate mophetil; *RTX* rituximab; *CYC* cyclophosphamide; *MTX* methotrexate; *MiTX* mitoxantrone; *CS* corticoids; *IVIG* intravenous immunoglobulins; *MS-DMD* Multiple Sclerosis-disease modifying drugs*; IM* intramuscular*; SC* subcutaneous

Table S2. Characteristics in patients with MOG antibodies and Multiple Sclerosis

| **Patient Id.** | **Sex** | **Age at onset, y** | **Relapses** | **First brain MRI** | **Spinal cord MRI at first episode** | **Lumbar puncture** | |  |
| --- | --- | --- | --- | --- | --- | --- | --- | --- |
|  |  |  |  |  |  | **OCB Cells/mm^3^** | | **^d^Binding index** |
| 1.MS | F | 21 | ON (x3) | ^a^MS-like | Normal | Positive | 9 | Intermediate |
| 2.MS | F | 34 | TM (x6) | ^b^MS-like | Short thoracic lesion | Positive | 0 | Low |
| 3.MS | F | 22 | TM (x2) | ^c^MS-like | Multifocal lesions along the SC | Positive | 147 | Intermediate |

*MS* multiple sclerosis; *F* female; *y* years; *MRI* magnetic resonance imaging; *OCB* oligoclonal bands; *TM* transverse myelitis; *LETM* longitudinally extensive transverse myelitis; *SC* spinal cord; *C* cervical; *D* thoracic

^a^Brain MRI fulfilling Barkhof criteria but some lesions.

^b^Brain MRI fulfilling Barkhof criteria but some lesions were fluffy with demarcated borders.

^c^Swanton and Paty criteria

^d^An estimation of MOG-Ab titre based on median fluorescent intensity in transfected versus untransfected cells (Cobo-Calvo, A et al. J Neurol 2019; 266:806-81).

Table S3. Comparison of baseline possible confounders between treated and non-treated patients in the original sample

| **Possible confounders** | **Non-treated patients,**  **n=59** | **Treated patients,**  **n=57** | **Type I-IS,**  **n=40** | **^a^p- value** | **^b^p- value** |
| --- | --- | --- | --- | --- | --- |
| Females, n (%) | 30 (50.9) | 33 (57.9) | 22 (55.0) | 0.446 | 0.685 |
| Age at onset, y mean (SD) | 34.7 (12.24) | 36.7 (13.05) | 38.5 (13.2) | 0.396 | **0.141** |
| Caucasian, n (%) | 57 (96.6) | 54 (94.7) | 37 (92.5) | 0.619 | 0.359 |
| Time between onset and first relapse, m mean (SD) | 31.3 (44.7) | 26.8 (45.6) | 24.5 (44.0) | 0.597 | 0.457 |
| Phenotype at onset, n (%)  ON | 44 (74.9) | 35 (61.4) | 25 (62.5) | **0.066** | **0.197** |
| Myelitis | 7 (11.9) | 16 (28.1) | 9 (22.5) |  |  |
| ON & myelitis | 2 (3.4) | 4 (7.02) | 4 (10.0) |  |  |
| Encephalopathic/  Brainstem S. | 6 (10.2) | 2 (3.5) | 2 (5.0) |  |  |
| EDSS at onset, mean (SD) | 2.6 (1.70) | 3.3 (1.88) | 3.0 (1.7) | **0.084** | **0.242** |
| Abnormal brain MRI,  at onset, n (%) | 13 (22.0) | 13 (22.8) | 8 (20.0) | 0.995 | 0.873 |
| Country of provenience, France, n (%) | 38 (64.4) | 44 (77.2) | 33 (82.5) | **0.130** | **0.050** |

^a^Differences between treated and non- treated patients

^b^Differences between type I-immunossuppressants treated patients and non-treated patients

For continuous and categorical variables t-test and chi-square were used, respectively.

*y* years; *m,* months*, SD* standard deviation*, ON* optic neuritis*, Brainstem S* brainstem Syndrome, *EDSS* Expanded Disability Status Scale*, MRI* magnetic resonance imaging, *IS* immunossuppressants

Table S4. Comparison of baseline possible confounders between treated and non-treated patients in the pseudo-population.

|  | **Propensity score weighting between non-treated and treated patients** | | | **Propensity score weighting between non-treated and Type I-IS treated patients** | | |
| --- | --- | --- | --- | --- | --- | --- |
| **Possible confounders** | **Non-treated patients,**  **n=59** | **Treated patients,**  **n=57** | **^a^Standardized differences** | **Non-treated patients,**  **n=59** | **Type I-IS,**  **n=40** | **^b^Standardized differences** |
| Females, % | 51.4 | 49.9 | 0.030 | 49.3 | 42.5 | 0.095 |
| Age at onset, y mean (SD) | 34.8 (12.63) | 35.3 (12.77) | 0.039 | 36.2 (13.03) | 35.7 (13.02) | 0.038 |
| Caucasian, n (%) | --- | --- | --- | --- | --- | --- |
| Time between onset and first relapse, mean (SD) | --- | --- | --- | --- | --- | --- |
| Phenotype at onset, %  ON | 68.2 | 69.2 | 0.021 | 71.1 | 73.4 | 0.028 |
| Myelitis | 20.4 | 19.9 | 0.012 | 14.9 | 14.3 | 0.017 |
| ON & myelitis | 4.5 | 5.2 | 0.028 | 6.06 | 6.08 | 0.001 |
| Encephalopathic/  Brainstem S. | 6.9 | 5.7 | 0.047 | 8.0 | 6.2 | 0.024 |
| EDSS at onset, mean (SD) | 2.9 (1.96) | 2.9 (1.73) | 0.035 | 2.74 (1.84) | 2.75 (1.56) | 0.005 |
| Abnormal brain MRI,  at onset | --- | --- | --- | --- | --- | --- |
| Country of provenience, France, % | 71.9 | 71.6 | 0.004 | 72.1 | 70.3 | 0.031 |

*Standardized differences for continuous and categorical variables were calculated as reported elsewhere.^18^

^a^Standardized differences between treated and non- treated patients

^b^Standardized differences between type I-immunossuppressants treated patients and non-treated patients

*y* years, *SD* standard deviation*, IS* immunosuppresants, *ON* optic neuritis*, Brainstem S* brainstem Syndrome*; EDSS* Expanded Disability Status Scale*; MRI* magnetic resonance imaging

| **Patient Id.** | **Combined drugs/**  **Time under combined therapy** | **Analysis 1** | **Analysis 2** |
| --- | --- | --- | --- |
| 4 | CS and AZT/  23 months | **Both IS non-included**  Both initiated treatment before first relapse | **Both IS non-included**  Time pre-treatment < 6 months |
| 20 | CS and MMF/  12 months | **MMF included. CS non included**  MMF included in the analysis  CS was initiated after MMF | **Both IS included** |
| 60 | CS and RTX/  6 months | **CS included. RTX non-included**  CS included in analysis  RTX < 6 months on treatment | **Both IS non-included**  Time pre- treatment < 6 months |
| 62 | CS and MMF/  4 months | **CS included. MMF non-included**  CS included in the analysis  MMF < 6 months on treatment | **Both IS non-included**  CS time pre- treatment < 6 months  MMF < 6 months on treatment |
| 63 | CS and MTX/  43 months | **CS included. MTX non included**  CS included in the analysis  MTX was initiated after CS | **Both IS included** |

Table S5. Patients who received combined drugs and reasons for inclusion/exclusion in different analyses

*IS immunosuppresants, CS corticosteroids, AZT azathioprine, MMF mycophenolate mophetil, RTX rituximab, MTX* methotrexate
