# Supplementary figures and images for: Evaluation of treatment response in adults with relapsing MOG-Ab-associated disease
Source: J Neuroinflammation. 2019 Jul 2;16:134. doi: 10.1186/s12974-019-1525-1 (PMC6607517; doi:10.1186/s12974-019-1525-1)

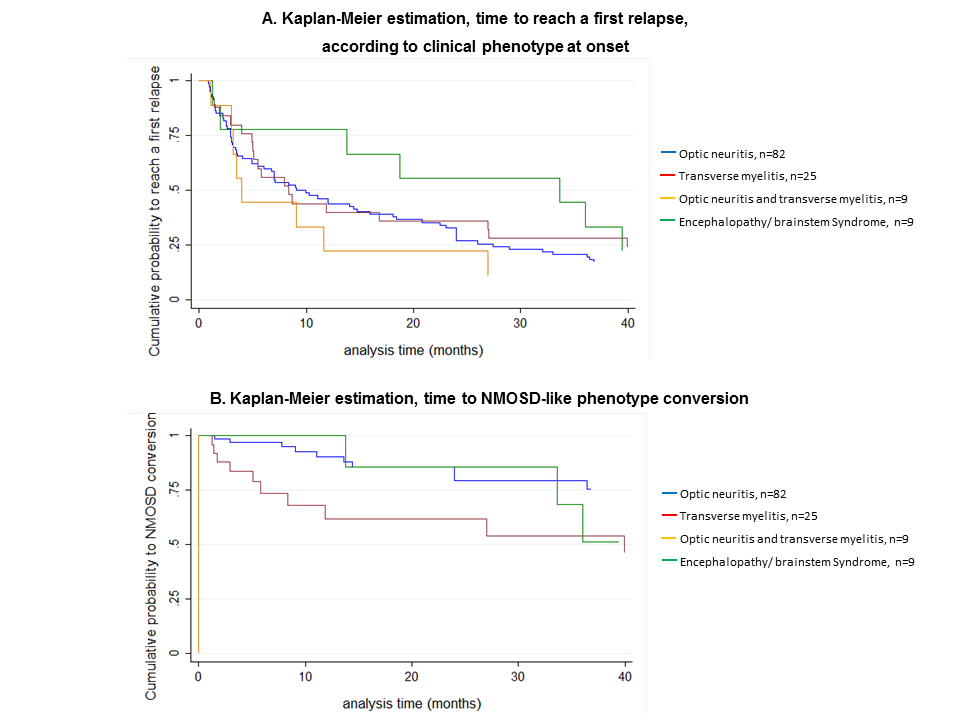

Supplement: Supplementary file 2 — Figure S1. (A) Kaplan- Meier estimation of time to first relapse, according to clinical phenotype at the onset. The 2-year risk of the first relapse was 70.7% (95%CI 60.7–80.1) for optic neuritis (reference), 64% (95%CI 45.8–81.8; Log-rank p = 0.589) for transverse myelitis, 77.8% (95%CI 48.7–96.6; Log-rank p = 0.458) for optic neuritis and transverse myelitis, and 44.4% (95%CI 19.6–79.6; Log-rank p = 0.617) for encephalopathy/brainstem syndrome. (B) Time to NMOSD-like phenotype conversion according to clinical phenotype at the onset. The 2-year risk to NMOSD-like phenotype conversion was 17.3%; 95%CI, 8.9–36.4 for optic neuritis (as a reference), 38.2% (95%CI, 20.8–63.0; Log-rank p = 0.032) for transverse myelitis, 14.3% (95%CI, 2.14–66.6; Log-rank p = 0.199) for encephalopathy/brainstem syndrome; 100% relapsed at onset (Log-rank p < 0.001) for optic neuritis and transverse myelitis (TIF 93 kb) [file 12974_2019_1525_MOESM2_ESM.tif]
